# Treating anxiety after stroke (TASK): the feasibility phase of a novel web-enabled randomised controlled trial

**DOI:** 10.1186/s40814-018-0329-x

**Published:** 2018-08-14

**Authors:** Ho-Yan Yvonne Chun, Alan J. Carson, Martin S. Dennis, Gillian E. Mead, William N. Whiteley

**Affiliations:** Centre for Clinical Brain Sciences, University of Edinburgh, Chancellor’s Building, 49 Little France Crescent, Edinburgh, Midlothian, EH16 4SB UK

**Keywords:** Telemedicine, Web-enabled, Cognitive behavioural therapy, Stroke, Anxiety, Wearable

## Abstract

**Background:**

Anxiety affects a quarter of strokes. It can be disabling even after mild stroke and transient ischaemic attack (TIA). It is not feasible to deliver conventional psychological therapies to the large population of anxious stroke and TIA patients. We are testing the feasibility of a web-enabled randomised controlled trial (RCT) to compare an individualised telemedicine cognitive behavioural therapy (CBT)-based intervention with a self-guided web-based relaxation programme. This study aims to evaluate the feasibility of novel trial procedures and the delivery of the TASK interventions in stroke and TIA patients.

**Methods:**

We aim to recruit 40 community-based stroke and TIA patients experiencing anxiety at least 1 month post-discharge in Lothian, Scotland. We will assess the (1) recruitment number per month; (2) percentage completion of electronic consent; (3) time taken for remote eligibility confirmation; (4) percentage completion of follow-up surveys: modified Rankin scale, EuroQol-5D5L, 7-item generalised anxiety disorder, Patient Health Questionnaire-2 and modified fear questionnaire; (5) data capture of intervention fidelity and (6) use of actigraph smartwatches to obtain continuous data of rest/activity.

**Discussion:**

The current study will provide feasibility data on streamlined web-enabled trial procedures and the use of smartwatches to obtain objective measures in stroke and TIA patients, offering potential for large efficient RCTs to be conducted centrally and remotely with far fewer resources in the future. This study will inform further refinements of the TASK interventions before evaluation in a definitive RCT.

**Trial registration:**

Clinicaltrials.gov NCT03439813. Retrospectively registered on 20/2/2018.

**Electronic supplementary material:**

The online version of this article (10.1186/s40814-018-0329-x) contains supplementary material, which is available to authorized users.

## Background

There are more than 100,000 strokes per year and 1.2 million stroke survivors in the UK [1]. Anxiety affects a quarter of stroke patients [2], equivalent to around 25,000 patients per year. Anxiety is associated with dependence, poorer quality of life and restricted participation in work and social activities after even mild stroke and TIA [3].

### Phobic and generalised anxiety

Anxiety is a universal emotion that helps people adapt to changing situations. However, it can become maladaptive when anxiety becomes pervasive or out-of-proportion to the danger posed by a situation. When maladaptive anxiety starts to interfere with a person’s occupational or social functioning, it is considered an anxiety disorder. Anxiety can be broadly divided into two clinical subtypes—phobic and generalised. Phobic anxiety is characterised by a disproportionate fear of well-defined situations or stimuli and marked avoidance of those situations [4]. By contrast, generalised anxiety disorder is diffuse and unremitting, characterised by persistent and multiple worries, e.g. finances, health and an inability to stop worrying [4]. In our recent prospective cohort, we found phobic disorder to be the predominant anxiety subtype after stroke and TIA [3].

### What are stroke patients anxious about?

Patients with anxiety disorder reported more avoidance in agoraphobia-related situations, e.g. going out alone, going to crowded places and travelling on public transport; social situations and specific situations, e.g. physical exertion, having sex, being alone at home and activities related to fear of having a headache, another stroke or a fall [3]. In our recent study, we found that the fear of stroke recurrence is the most commonly reported anxiety-provoking thought post stroke/TIA. This fear appeared to have led to differential behaviours in our patients. In some, this anticipatory anxiety brought about a desire for better health and increased positive health behaviours, e.g. complying with medications and doctor’s advice on lifestyle, while others developed a grossly distorted view of their risk of stroke recurrence despite adhering to secondary prevention [3]. These patients feared having a debilitating stroke on a regular basis, perpetuating maladaptive avoidance of daily situations. Both avoidant behaviours and distorted thinking are targets for a CBT-based intervention.

### Predictors for anxiety after stroke/TIA

Younger people and those with a previous history of anxiety or depression are more likely to develop anxiety after stroke [3, 5]. Longitudinal data suggested anxiety post stroke could last up to 10 years [6].

### Barriers to accessing CBT in stroke and TIA

Inadequate service provision in psychological care post stroke across the UK is evident from the reports of the Sentinel Stroke National Audit Programme in England, Wales and Northern Ireland [7] and the latest Royal College of Physicians Stroke Guideline [8]. The demand for better access to psychological care post stroke has consistently been echoed by surveys and qualitative research of patients, carers and health professionals, and through charitable organisations representing stroke patients [9–11]. Delivering CBT in a conventional face-to-face format to all patients with anxiety post stroke/TIA is not feasible in clinical practice given the high prevalence of anxiety in this population and limited resources. In Scotland alone, a quarter of stroke patients equate to 2000–3000 patients per year. Our stakeholder activity involving the clinical leads of stroke and clinical psychology services from across Scotland (Additional file 1) agreed that this demand was unlikely to be met by primary care providers alone, nor the traditional paradigm of referring patients for face-to-face psychotherapy delivered by highly-trained specialists. The shortage of highly trained staff and geographical variation was unlikely to be resolved in the near future.

Other potential barriers to accessing psychological therapy include the inability to attend face-to-face appointments due to physical immobility and social stigma attached to seeking psychological treatment. Reluctance to travel in agoraphobia and the fear of being judged in social phobia are diagnosis-specific barriers to attending face-to-face sessions. People with social phobia symptoms were three times more likely to report a fear of what others might think or say about them if they sought psychological treatment compared to people with other anxiety disorders [12]. To date, randomised controlled trials (RCT) of guided internet-delivered CBT for anxiety and depressive disorder in general adults have shown good patient adherence and satisfaction [13].

### Potential of a telemedicine CBT-based intervention

Telemedicine could offer a way to overcome the barriers in accessing psychological therapy by treating patients remotely while maintaining an individualised therapist-patient alliance that is integral to CBT. Meta-analyses demonstrated guided internet-based CBT to be superior to waitlist control and as efficacious as face-to-face CBT in treating anxiety disorders or depression in general adults, with face-to-face CBT spending seven times more therapist time than guided internet-based CBT [13, 14]. It is not yet certain whether these findings could be generalised to stroke and TIA patients, who tend to be older, have neurological deficits and medical co-morbidities. The effect of such interventions on patient outcomes after stroke/TIA needs to be evaluated in RCTs.

Delivering CBT remotely via telemedicine would enable centralisation of resources for staff, training, quality monitoring and cross-covering of different geographical areas, reducing travel time for therapists as well as patients. Digital content and treatment approaches in a telemedicine intervention could be updated and refined easily, thus, making use of the latest best evidence. The provision, maintenance and updating of digital content could be commissioned or delivered via charitable organisations. Organisation delivering the intervention can continue to invest in research and development to utilise the latest technology to deliver the treatment content at lower costs. At present, it is not yet clear whether telemedicine-delivered CBT is cost effective in stroke and TIA patients.

### TASK intervention development

**Fig. 1:**
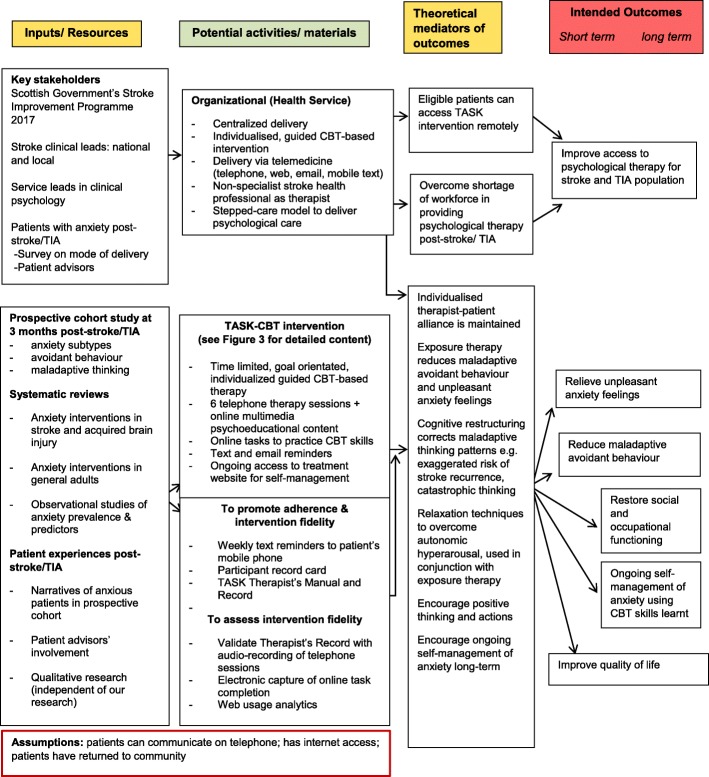
Modelling processes and outcomes of TASK-CBT

We developed the treating anxiety after stroke (TASK) CBT intervention following the UK Medical Research Council’s framework for complex intervention development [15], elaborated by the Six essential Steps in Quality Intervention Development (6SQUiD) [16]—a formal methodology using a systematic, logical and evidence-based approach for complex intervention development. Using this approach, we employed existing research findings, our original research and stakeholders’ activities in developing TASK-CBT, reported in detail in Additional files 1 and 2. We modelled the processes and outcomes of the TASK-CBT intervention in a logic diagram in Fig. 1. Our current study represents the process evaluation and feasibility testing in a small scale before TASK-CBT can be evaluated in a definitive RCT.

### Conducting an RCT entirely remotely by applying information technology

A definitive RCT needs to recruit and retain large number of patients to provide sufficient power to detect a clinically relevant treatment effect. Web-enabled and automated trial procedures can centralise processes and resources, offering the potential for scaling up an RCT with far fewer resources and costs than conventional procedures. For instance, one of the major costs of traditional multicentre trials is the setting up of multiple centres which includes establishing legal contract between the sponsor and local sites, identification and training of local staff, site initiation visits, monitoring, local closeout and archiving. Adopting a novel centralised system could avoid the need for these and streamline a large trial. Feasibility of remotely performed trial procedures need to be tested in stroke and TIA patients: online self-screening and recruitment, remote eligibility confirmation, electronic informed consent, remote intervention delivery, automated self-reported outcome measures and data capture of intervention fidelity.

### Use of smartwatches to collect continuous data on activity/rest in an RCT

Anxious patients after stroke showed higher levels of avoidant behaviour across a range of situations, e.g. going out alone, physical exertion and social situations compared with those who were not anxious [3]. Disturbed sleep is a feature common to both anxiety and depression. RCTs of psychological interventions have conventionally relied on self-reported outcomes using questionnaires. This requires effort from participants. Non-responders (attrition) can bias results and reduce the power of the study, resulting in a waste of valuable research resources. A wrist-worn smartwatch that records actigraphy (non-invasive method of monitoring rest/activity cycles) continuously could provide data on activity and sleep, offering the potential of measuring objective outcomes throughout the entire RCT with minimal patient effort. The feasibility of long-term continuous monitoring using this method has not previously been tested in RCTs of psychological intervention or RCTs of stroke/TIA patients [17]. The requirement for participants to return smartwatches during an RCT could pose an attrition issue which we will explore in the current study.

### Objectives

This study aims to evaluate the feasibility of (i) novel web-enabled trial procedures, (ii) the TASK-CBT intervention and (iii) actigraph smartwatches to collect continuous data throughout the entire trial in stroke/TIA patients.

## Methods

We report the protocol of the TASK feasibility RCT in accordance with the SPIRIT checklist [18] Additional file 3). Description of TASK-CBT adheres to items on the TiDier (template for intervention description and replication) checklist [19] (Additional file 4). The trial protocol is registered at ClinicalTrials.gov (NCT03439813).

### Trial design

**Fig. 2:**
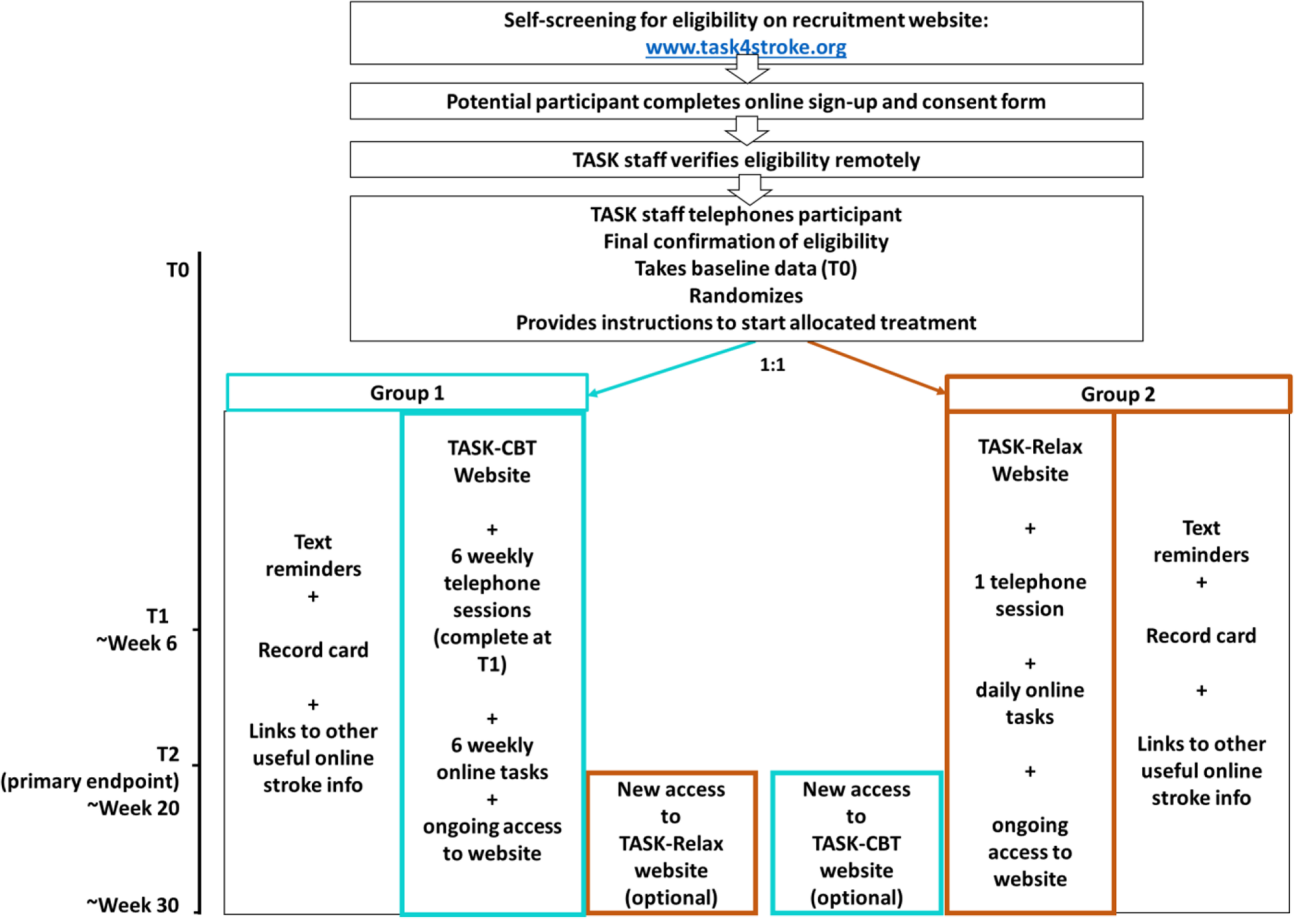
TASK feasibility trial schematic

The TASK feasibility trial is a parallel two-armed RCT comparing TASK-CBT with TASK-Relax. Figure 2 illustrates the participant timeline in a schematic.

#### Information technology used in the design of the TASK feasibility RCT

We designed the TASK trial procedures using Research Electronic Data Capture (REDCap), a secure web-based database management application [20]. We created three websites, one for recruitment (www.task4stroke.org) [21], one for the TASK-CBT intervention and one for the comparator TASK-Relax using an industry-led website builder. Using REDCap, we embedded data collection forms in our websites for self-screening for eligibility, informed consent and online tasks for TASK-CBT. Usage of all websites is monitored with Google Analytics. In our smartwatch sub study, we are testing the feasibility of GENEActiv Original smartwatch [22], a device designed primarily for research with validation data [22]. Continuous data (triaxial accelerometry, temperature, light) are recorded and can be downloaded from each device for analysis of physical activity and sleep. No data are stored on a commercial ‘cloud’, and no patient identifiable data are stored on the device.

#### Patient population—inclusion and exclusion criteria

We aim to recruit 40 community-based residents within NHS Lothian (United Kingdom postcodes EH and FK1) who are aged 18 or above, with a diagnosis of stroke or TIA (probable, definite or ocular), at least 1 month after being discharged to the community from clinic or hospital ward. Participants need to have capacity to give informed consent, be able to communicate in English on the telephone, have internet access and report at least one positive response on our 6-item anxiety screening questions (Additional file 5). These items were derived from the GAD-7 [23] and modified fear questionnaire [3, 5] using psychometric techniques including factor analysis, analysis of internal consistency and Mokken scaling [Chun, H.-Y.Y., et al. Deriving the 6-item anxiety screening questions for a randomised controlled trial in stroke. Unpublished 2018].

We exclude people already taking part in a clinical trial of treatment intended to improve psychosocial outcomes post stroke.

#### Recruitment methods

The TASK recruitment website: www.task4stroke.org [21] is publically accessible, where participant information is available via a video or a readable format. Interested potential participants can complete the ‘Sign Up and Consent Form’ on the website. We disseminate the website address as widely as possible amongst patients, community stroke nurses, stroke physicians, stroke rehabilitation therapists and stroke charities using printed ‘business cards’, flyers and social media. We offer trial information to stroke and TIA patients during their one-month telephone follow-up by a stroke clinician as part of routine stroke care. In addition, we sent postal invitations to eligible participants identified from the NHS Lothian stroke audit registry retrospectively.

On receiving the completed ‘Sign Up and Consent Form’, the TASK research team verifies the eligibility and identity of the potential participant using electronic health records and over the telephone with the participant within five working days. Baseline data are immediately collected at this point (T0), followed by randomisation.

#### Intervention and comparator

We designed the TASK-CBT intervention to be delivered via telemedicine—telephone, website, email and mobile text. This enables remote delivery of the intervention while preserving the individualised therapeutic alliance between the therapist and the patient that is integral to CBT. The TASK-CBT intervention represents a low-intensity, guided self-help psychological intervention in a stepped care model [24]. A health professional with experience working with stroke patients, e.g. stroke nurse, stroke physician delivers TASK-CBT under the supervision of a specialist, e.g. psychiatrist or clinical psychologist.

#### Delivery of ‘active ingredients’ of TASK-CBT by telephone and web

**Fig. 3:**
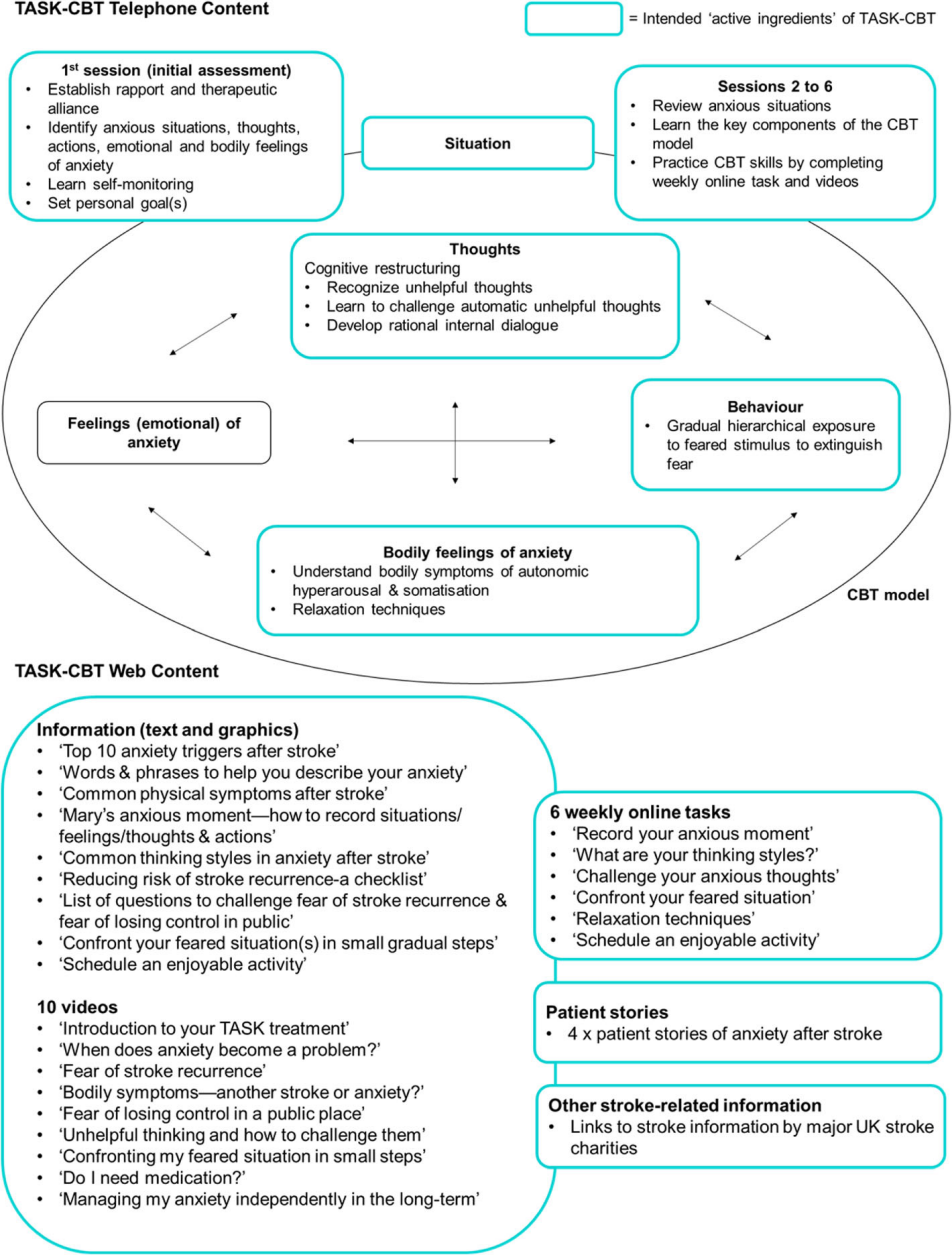
TASK-CBT content

We summarise the key ‘active ingredients’ of the TASK-CBT intervention in Fig. 3. In brief, the TASK therapist delivers a course of six individualised telephone CBT sessions, 35–45 min each, at least 1 week apart, with the use of an electronic TASK therapist’s manual and record. Each session is supplemented by the prescription of an online task and one or more of the psychoeducational videos on the TASK-CBT treatment website. To encourage adherence, we send a mobile text to remind participant to complete the online task each week and to use the treatment website as much as possible via a computer, tablet or smartphone.

#### Active comparator: TASK-Relax

TASK-Relax is a web-based self-guided relaxation programme. Relaxation therapy is a commonly used comparator in RCTs of CBT in psychiatry research [25]. The TASK-Relax website consists of an introductory video, followed by five relaxation tasks: (i) audio- and visually-guided breathing exercise, (ii) relaxing imagery and sounds, (iii) music for relaxation, (iv) audio-guided progressive muscle relaxation and (v) a selection of sounds of nature. All relaxation videos/audios on the TASK-Relax website are also publically available on YouTube. Participants allocated to TASK-Relax receive instructions to try out all of the relaxation exercises, then select their favourite one(s) to practice daily, for at least 5 min throughout their trial participation.

#### Components common to both groups

Participants of both arms receive weekly mobile text reminder and a participant record card to record progress and completion of follow-up surveys. Data collection occurs at T1(week 6) and T2 (week 20, primary endpoint) via emailed links to self-completed electronic surveys. Once the follow-up survey at T2 is complete, we offer all participants access to the website given to the other group for a further 10 weeks.

#### Concomitant care and interventions

Concomitant standard clinical care and interventions (pharmacological or non-pharmacological) for anxiety or mood disorders, e.g. antidepressants, benzodiazepines are permitted and recorded in our follow-up surveys.

#### Randomisation and allocation concealment

A member of the research staff not involved in conducting the TASK trial generates a permuted block randomisation sequence with random block sizes using STATA14 [26]. The sequence is uploaded to the in-built randomisation module within REDCap, which is inaccessible to the TASK researcher enrolling participants. Once baseline data collection is complete, the TASK researcher randomises the participant and emails him/her the allocated treatment website address and login details. Participants receive telephone instructions on commencing the allocated treatment. Participants allocated to TASK-CBT also receive an appointment for their first telephone session.

#### Masking

The TASK researcher (HYC) enrolling participants and delivering the allocated intervention is not blinded to the treatment group assignment. The TASK researcher informs participants that they will be randomly allocated to one of two anxiety interventions by the computer and receive the login details to their allocated website via email. Participants are masked to the contents and type of anxiety intervention allocated to the other group. This method attempts to mask participants to our hypothesis that one treatment is superior to the other.

#### Sub study of using a smartwatch to measure actigraphy continuously

In a sub study embedded within the TASK trial, all TASK participants are invited to wear the actigraph smartwatch. Once consented, the smartwatch is posted to the participant with simple care instructions. At 2 months, the battery of the smartwatch will run out. With the participant’s agreement and on the safe return of the first smartwatch, a second smartwatch will be sent to the participant, so he/she can wear it for the rest of the trial. All smartwatches are returned using prepaid special delivery envelopes.

#### Feasibility outcomes

The current trial assesses the feasibility of the TASK-CBT intervention and the technology-enabled trial procedures. Feasibility data collected: recruitment (number per month), percentage of completed consent (online or by post), time taken to complete remote eligibility confirmation via electronic health records (date of randomisation–date of ‘Sign Up and Consent Form’ received), percentage drop out after fewer than three telephone TASK-CBT sessions and self-completion of electronic follow-up surveys at T1 and T2 (percentage of completed surveys).

We define a lack of feasibility of TASK-CBT and the current trial design as (1) recruitment number of < 2 per month, (2) > 50% of TASK-CBT patients dropping out after fewer than three telephone sessions, (3) > 10% non-completion of follow up surveys at T2 and (4) participants reporting harm from the intervention.

Treatment relevant outcomes (Table 1) include the modified Rankin Scale for dependence [27], EuroQol-5D5L [28], 7-item generalised anxiety disorder [23], modified fear questionnaire (FQ) [3, 29], Patient Health Questionnaire-2 (PHQ-2) [30] and a single question to elicit concurrent treatment for mood or anxiety. A user feedback survey automatically follows the T2 follow-up survey.

**Table 1:** Treatment relevant outcomes

	T0 Baseline (pre-randomisation)	T1 follow-up (~ week 6)	T2 follow-up primary endpoint (~ week 20)
Demographics	*		
Diagnosis (stroke or TIA)	*		
Past history of anxiety or depression	*		
Medications for anxiety or mood	*		
mRS	*	*	*
EQ5D5L-VAS	*	*	*
GAD-7	*	*	*
PHQ-2	*	*	*
Modified FQ	*	*	*
Single question on concurrent treatment for mood or anxiety (drug or non-drug)	*	*	*
User feedback survey			*
Sub study of wearing a smartwatch			
Smartwatch for measuring rest/activity	Continuous monitoring throughout the trial from T0 to T2		

*mRS* modified Rankin Scale, *EQ5D5L-VAS* EuroQol-5D5L-Visual analogue score, *GAD-7* 7-item generalised anxiety disorder questionnaire, *PHQ-2* 2-item Patient Health Questionnaire, *FQ* fear questionnaire

#### Feasibility outcomes of the smartwatch sub study

We will assess the percentage of TASK participants who also consent to wearing the smartwatch, the duration of the smartwatch being worn by each participant using the data recorded on the smartwatch, percentage of participants of this sub study who agree to wear the smartwatch again after 2 months, percentage of participants who did not return smartwatch (attrition).

#### Assessing intervention fidelity and quality monitoring of therapist

The therapist for this feasibility trial is a stroke physician (HYC) who has received training on delivering CBT from and has ongoing supervision by a consultant neuropsychiatrist (AJC) at weekly meetings during the TASK trial. For intervention fidelity, we will assess (i) percentage agreement between the therapist’s record and transcripts of audio-recording of telephone sessions, assessed by a clinician independent of the study; (ii) automated data capture of online task completion; (iii) summary web usage data from Google Analytics.

#### Strategies to improve adherence in both arms

We post a participant record card to every participant and send weekly text reminders to their mobile phone throughout their participation. All participants will receive text and email reminders to complete the follow-up surveys at T1 and T2. Non-responders will receive further text and email reminders and a final phone call from a researcher blinded to the treatment allocation to complete unfilled online surveys over the telephone.

#### Discontinuation criteria

Participants are free to withdraw from the study at any point, or a participant can be withdrawn by one of the TASK trial investigators if he/she loses capacity during the study period.

#### Safety protocol

A trained medical doctor delivers the TASK-CBT intervention in the current feasibility trial. All participants have contact details of the TASK research team from the start of the trial. They are informed that if severe cases of anxiety or depression are identified during the trial, the TASK research team will liaise with their general practitioner to arrange appropriate care. Trial investigators will assess these cases and report as adverse events to the sponsor according to good clinical practice.

#### Data management

All study data are collected and managed using REDCap electronic data capture tools hosted at University of Edinburgh. REDCap (Research Electronic Data Capture) is a secure, web-based application designed to support data capture for research studies [20]. Identifiable data are only accessible by the TASK trial investigators and managing team of the REDCap database. Anonymised data from all smartwatch devices will be downloaded as binary files and sent to our collaborating data scientist at the Centre for Medical Informatics, Usher Institute, University of Edinburgh for analysis.

#### Data monitoring body

There is no data monitoring committee planned for this study.

#### Statistical analyses and power calculation

As this is a feasibility study, we did not perform power calculation. Feasibility outcomes will be summarised descriptively. All electronic items on the follow-up surveys must be scored to permit submission, preventing any missing values. Only the trial investigators have access to the final trial dataset. Actigraphy data from the smartwatch device will be sent anonymously for time-series analysis at the Centre for Medical Informatics, Usher Institute, University of Edinburgh.

## Discussion

Using innovative information technology, we designed a streamlined web-enabled trial to be tested for the first time in stroke and TIA patients: online self-screening and recruitment, remote eligibility confirmation, electronic informed consent, remote intervention delivery, automated self-reported outcome measures and data capture of intervention fidelity. Our trial design offers a number of potential advantages. For instance, a centralised model could enable recruitment of eligible patients anywhere in the country, or anywhere in the world without the need to have locally-based principal investigators or research staff. Larger sample sizes could be reached more easily without significantly raising extra costs. Potential participants with proof of identity and eligibility could sign up themselves regardless of their locations. This model of recruitment could empower patients to choose to participate in a clinical trial without being screened or ‘selected’ by local healthcare or research staff. Generalisability could be increased as a result. While this method of recruitment remains aspirational for clinical trialists at present, owing to the regulatory and administrative challenges in conducting multicentre RCTs, it is of vital importance that we continue to evaluate the feasibility of novel procedures that would increase the ease of recruiting participants to large-scale RCTs, regardless of where they live. A centralised system of intervention delivery could be easily scaled up but also quality assured—a particular challenge in clinical trials where many therapists are employed to deliver the intervention in different centres.

There has been limited use of wearable devices to assess therapeutic outcomes in RCTs in stroke patients and RCTs of psychological interventions [17]. We recently observed high levels of avoidant behaviour across a range of situations, e.g. going out alone, physical exertion and social situations in people experiencing anxiety after mild stroke and TIA [3]. An actigraph smartwatch that records continuous data on activity/rest throughout the entire RCT offers an easily scalable way of collecting objective measures in large clinical trials with minimal patient effort. This study will inform whether using smartwatches could reduce attrition in RCTs, thus reducing bias in trial results and avoiding waste of valuable research resources.

### Improving access to psychological therapy via a centralised model of delivery

Anxiety is one of the commonest neuropsychiatric complications post stroke or TIA. Psychological care post stroke/TIA is inadequate, and the traditional paradigm of delivering face-to-face CBT at clinic is not feasible for the large population of stroke and TIA patients owing to limited resources and other barriers related to anxiety after stroke, e.g. agoraphobia and social phobia. Innovative ways of delivering psychological intervention remotely to the patient’s home, e.g. individualised CBT via telemedicine could be a feasible model to treat the large population of stroke and TIA patients experiencing anxiety. If feasible, the TASK-CBT intervention could provide an exemplar model for delivering psychological intervention for a range of post-stroke psychological complications. Web-enabled features embedded in our TASK-CBT and TASK-Relax websites, e.g. data capture of completion of weekly online tasks and web usage analytics, provide novel ways of measuring intervention fidelity objectively—an important aspect of internal validity that can be challenging to assess and is infrequently reported in trials of complex intervention [31].

The TASK trial design has the potential to be applied in RCTs of any self-management or guided self-management interventions for a range of patient groups. Our design has the obvious limitation of excluding people who have no access to the internet, or those who do not have a minimum level of skills in information technology. This is likely to be overcome by the trend towards wider adoption of home broadband and user-friendly devices amongst seniors [32], and the encouragement and assistance provided by family members.

### Considerations for the next phase, TASK II RCT

The current trial represents feasibility testing of the TASK-CBT intervention and the technology-enabled trial procedures. This will lead to further refinements of intervention design and trial procedures. Our current trial does not inform the efficacy of TASK-CBT. A number of important aspects of the TASK trial design will need to be considered in the next phase, the TASK II trial, before proceeding to the definitive trial, TASK III.

#### Choice of primary outcome

In TASK II, we will determine an appropriate primary outcome measure to be used in the definitive trial. We will consider a range of candidate outcome measures of anxiety, functional independence, quality of life and participation. We will select the primary outcome based on the measure’s sensitivity to change/difference between groups, its practicality for use in large-scale RCT (to minimise attrition) and its clinical meaningfulness. Our observational data suggested that stroke/TIA patients with anxiety disorder reported a statistically significantly higher level of functional dependence on the modified Rankin Scale, a poorer quality of life on EurolQol-5D-5L and worse participation on a the Work and Social Adjustment Scale, compared to those without anxiety [3].

Patients after stroke/TIA can present, to varying degree, predominantly phobic, predominantly generalised, or mixed anxiety types. This poses an additional challenge as different anxiety measures are required to assess the two anxiety subtypes. It may be necessary to have different primary outcomes defined for different anxiety subtypes.

Other considerations include defining the responder’s status: for example, whether to use the minimal relevant change from baseline on an anxiety measure, a dichotomised outcome, or an absolute anxiety level post-intervention to assess between-group difference.

#### Choice of comparator

Our choice of using TASK-Relax, a web-guided relaxation programme as an active control was to avoid exaggerating the effect size—a known effect of using ‘waitlist’ or ‘treatment as usual’ as control condition in RCTs of psychological interventions [33]. We accept that TASK-Relax could have an effect on anxiety in the short-term, but our hypothesis is that TASK-CBT will have a long-term effect while TASK-Relax would not (at week 20). TASK II will provide information on the outcome measure and variability in the two groups, informing the minimally important difference for our power calculations.

#### TASK intervention design

We will refine the TASK-CBT intervention based on participants’ feedback from this current trial. The TASK therapist training materials will be developed based on our experience and the use of anonymised audio-recording transcripts. We will consider using audio recording for within-trial training, maintenance and enhancement of intervention fidelity in the next phase of TASK, where therapists will be employed to deliver the TASK-CBT intervention.

#### Cost effectiveness

As it is not yet clear whether TASK-CBT is cost-effective in stroke and TIA patients, health economics analysis of the TASK-CBT intervention using EuroQoL-5D-5L data will be conducted in the definitive RCT.

#### Efficient large-scale RCT using web-enabled procedures

Testing the feasibility of self-recruitment and remote eligibility confirmation in our local centre represents only the first step in finding novel efficient ways of recruiting patients to large-scale nationwide RCTs. We believe all patients should be empowered to self-recruit to an RCT that they are eligible to regardless of where they live. Verifying eligibility remotely using electronic health records across different health boards in RCTs could be a viable option in the future but at present remains aspirational due to administrative barriers.

### Trial status

Protocol version: AC17087 version 3, 1/12/2017.

Recruitment commenced on 17/1/2018. Estimated completion date 1/5/2018.

## Additional files

Additional file 1: TASK-CBT Intervention development using the 6SQuID approach. (PDF 725 kb)

Additional file 2: Report of patient involvement in TASK. (DOCX 463 kb)

Additional file 3: SPIRIT checklist items. (DOC 120 kb)

Additional file 4: TiDier template items. (DOCX 28 kb)

Additional file 5: Screening questions in ‘Sign up and consent form’. (PDF 173 kb)

Additional file 6: Research ethics approval letter. (PDF 134 kb)
