# Supplementary material for: Treating anxiety after stroke (TASK): the feasibility phase of a novel web-enabled randomised controlled trial
Source: Pilot Feasibility Stud. 2018 Aug 14;4:139. doi: 10.1186/s40814-018-0329-x (PMC6092858; doi:10.1186/s40814-018-0329-x)
Supplement: Supplementary file 1 — TASK-CBT Intervention development using the 6SQuID approach. (PDF 725 kb) [file 40814_2018_329_MOESM1_ESM.pdf]

Additional file 1

Treating anxiety after stroke (TASK) Intervention development

We report our TASK intervention development using the six essential steps in complex intervention development (6SQuID approach (Table 1)[1].

Table 1. The six essential steps in complex intervention development
1. Define and understand the problem and its causes<br>2. Clarify which causal or contextual factors are modifiable and have the greatest scope for change<br>3. Identify how to bring about change: theory of change<br>4. Identify how to deliver the change: theory of action<br>5. Test and refine on a small scale<br>6. Collect sufficient evidence of effectiveness to justify rigorous evaluation/ implementation

6SQUID Step 1. Define and understand the problem and its causes

There are more than 100,000 strokes per year, and 1.2 million stroke survivors in the United Kingdom[2]. Anxiety affects a quarter of stroke patients, equivalent to around 25, 000 patients per year. Anxiety is associated with dependence, poorer quality of life and restricted participation in work and social activities after even mild stroke and transient ischaemic attack (TIA)[3]. Anxiety is classified into two main clinical subtypes—phobic and generalized anxiety disorder (GAD), requiring different treatment approaches[4]. Phobic disorder e.g. agoraphobia, specific phobia, appears to be the predominant anxiety subtype post-stroke/TIA and can occur with or without GAD[3, 5]. Longitudinal data in stroke suggest that anxiety post-stroke can last up to 10 years[6]. When left untreated, anxiety disorder does not tend to resolve in non-stroke populations.

Phobic and generalized anxiety

Phobic anxiety is characterized by a disproportionate fear of well-defined situations or stimuli[4]. Exposure to the feared situation triggers unpleasant anxiety symptoms, accompanied by marked avoidant behaviour of that feared situation—the hallmark of phobic anxiety. Treatment of phobic disorders requires systematic, repeated, hierarchical exposure to the specific anxiety-provoking stimulus[7]. GAD is diffuse and unremitting, characterized by persistent and multiple worries e.g. financial security, health, and an inability to stop worrying[4]. Cognitive behavioural therapy techniques e.g. cognitive restructuring, problem solving and/ or selective serotonin-reuptake inhibitors (SSRI), benzodiazepines (in short term only) are effective at treating GAD[8, 9].

Pathophysiology of anxiety

Anxiety is the most extensively researched emotion in science. The study of anxiety and its pathological forms supports a multifactorial aetiology of clinical anxiety disorders—the complex interplay of biological (genetic, physiological), psychological, behavioural and social (environmental) factors. Studies of the sympatho-medullary pathway (in the fight-or-flight response), the hypothalamo-pituitary-adrenal axis, and the mapping of a complex neurocircuitry connecting structures such as the amygdala, prefrontal cortex, anterior cingulate cortex, hypothalamus etc. have improved our understanding of the neurobiology of anxiety[10, 11]. Studies of classical conditioning—a learning behaviour shared by animals and humans, and of maladaptive thinking (cognitive) patterns established the basis for cognitive behavioural therapy (CBT), now a first line treatment for a range of anxiety disorders supported by randomised controlled trial (RCT) evidence[8, 12].

Fear conditioning as a mechanism for phobic disorder—the basis for exposure therapy

Fear can be acquired and extinguished via 'classical conditioning', also known as 'learning by association'. Ivan Pavlov in the late nineteenth century discovered that dogs learnt by classical conditioning—by repeatedly pairing the ringing of a bell (a neutral stimulus) with

food (a natural stimulus of salivation), his dogs became conditioned to salivate on hearing the bell, even in the absence of food[13]. In 1920, John Watson and Rosalie Raynor demonstrated that a nine-month old infant, known as little Albert, could be conditioned to exhibit fear to a previously neutral stimulus (a white fluffy rat) by repeatedly presenting the rat with a naturally aversive stimulus—a loud hammering sound[14]. His experiments also demonstrated that phobic fear of one stimulus could generalize across a range of similar situations—little Albert's fear extended to other similarly fluffy objects, including a fur coat and a Santa Claus mask[14]. Shortly following this, Mary Jones went on to apply the same principles of classical conditioning to extinguish the phobia of rabbits in a child named Peter, by repeatedly presenting him a rabbit together with his favourite candy while gradually bringing the animal closer to him[15]. Classical conditioning, complemented by operant conditioning[16, 17], in which reward led to positive reinforcement of a behaviour, are learning behaviour theories fundamental to exposure therapy—a behavioural therapy. In systematic desensitization, the present form of exposure therapy developed by Joseph Wolpe[18], phobic individuals confront their defined feared situation in gradual hierarchical steps. Through conditioning and habituation, the unpleasant feelings of anxiety associated with that situation gradually diminish. By encouraging the individual to confront their feared situation, exposure therapy breaks the vicious cycle of maladaptive escape/ avoidant behaviour, which, prior to treatment, had been reinforced by the pleasant sense of relief and comfort that avoidance brings. Anxiety feelings are emotionally distressing and the maladaptive behaviour that follows leads to impaired social and occupational functioning in phobic individuals. Exposure therapy, usually delivered as part of CBT, is an effective treatment for phobic disorders in the non-stroke populations[7, 19], and yet it has never been evaluated in stroke patients with anxieyt[20].

Maladaptive thinking as a mechanism for anxiety—the cognitive model

In the cognitive model established by Aaron Beck and Albert Ellis in the 1960s, individual's feelings of anxiety arise from maladaptive thoughts—misconception, distortions (overgeneralization, exaggeration) and faulty assumptions about himself and/or his world. Cognitive therapy guides the individual to i) recognize these maladaptive thoughts, ii) helps him appraise them objectively by testing the validity of these thoughts with rules of evidence, logic or alternative explanation in a process known as cognitive restructuring[21]. Cognitive and behavioural models merged in the 1970s and CBT became widely used to treat a range of mental health disorders. In recent decades, RCTs established an evidence base for the effectiveness of CBT in anxiety disorders[19].

What provokes the maladaptive behaviour and maladaptive thinking patterns in stroke patients?

Most people perceive stroke to be a sudden and potentially life-threatening event that carries a risk of recurrence. Fear conditioning appears to be a mechanism that contributes to anxiety after stroke. We found in our recent prospective cohort study, that phobic disorder was the predominant anxiety subtype after stroke or TIA[3]. Participants with anxiety reported significantly higher levels of phobic avoidance on the modified Fear Questionnaire compared to participants without anxiety disorder. Phobic avoidance was present in 'agoraphobic-related' situations: going alone far from home, going into crowded shops, travelling alone or by bus; 'social' situations: being watched or stared at, eating and drinking with other people, being criticized; 'other specific' situations: physical exertion, having sex, being alone at home[22]. The fears of stroke recurrence, falling, and headaches/ bodily sensations were reported as anxiety-provoking in this cohort. Our psychiatric interviews suggested that anxious participants became conditioned to experience the unpleasant feelings of anxiety/ fear in otherwise 'neutral' situations e.g. going out alone, taking the bus, going into a crowded shop, taking a shower, with ensuing avoidance of these situations. The predominant

thinking pattern in these situations was the possibility of stroke recurrence, or other similarly dangerous/ embarrassing mishaps. The perceived risk of this danger appeared exaggerated in our anxious participants.

Patient involvement and other qualitative evidence

In our patient involvement work, reported in detail in Additional file 5, we asked three patient advisors who experienced anxiety post-stroke to elaborate further on their anxiety issues and to co-produce the content of our intervention. The fear of *'completely losing control in a public place in case of another stroke'* was the most frightening thought for all of them. They feared not only for their own safety, but also how other people would react to them losing control of their body or speech. In addition, the experience of recurrent non-specific bodily symptoms e.g. headache, stabbing pains, accompanied by the uncertainty of whether these symptoms represented stroke recurrence provoked a great deal of anxiety in our patient advisors.

Individual and systematic review of qualitative research[23-25] conducted independently of our work, complemented our own findings. Patients and carers expressed themes and gave narratives about the fear of stroke recurrence[23], insecurity about going out and going on the bus[24], fear of falling[26], and the feeling of body not under own control[23].

Risk factors for anxiety post-stroke

Younger age and having a previous history of anxiety are the most consistent predictors for developing anxiety post-stroke[22, 27]. Co-morbid depression is also common in anxiety after stroke[1]. Systematic review did not find sufficient evidence to support any association with potentially modifiable psychological factors such as locus of control, coping style or confidence that have been implicated in anxiety in other disease populations e.g. multiple sclerosis[28, 29].

6SQUID Step 2 Clarify which causal or contextual factors are modifiable and have the greatest scope for change

Our 6SQUID Step 1 suggests that maladaptive avoidance and thinking patterns (e.g. exaggerated risk of stroke recurrence) are frequently reported in people with anxiety post-stroke/TIA, and so could be targeted by CBT. Considering the existing RCT evidence on CBT's effectiveness in non-stroke populations, adapting CBT approaches, including exposure therapy to treat stroke patients with anxiety should offer the greatest scope for change.

6SQUID Step 3 Theory of change

Aside from maladaptive avoidance and thinking patterns, there are other potential factors, whether theoretical and evidence-supported, that may contribute to anxiety after stroke. We summarized the list of potential contributory factors to anxiety after stroke in our theory of change diagram (Figure 1). The TASK intervention targets the factors in red.

Figure 1. Theory of change in anxiety after stroke

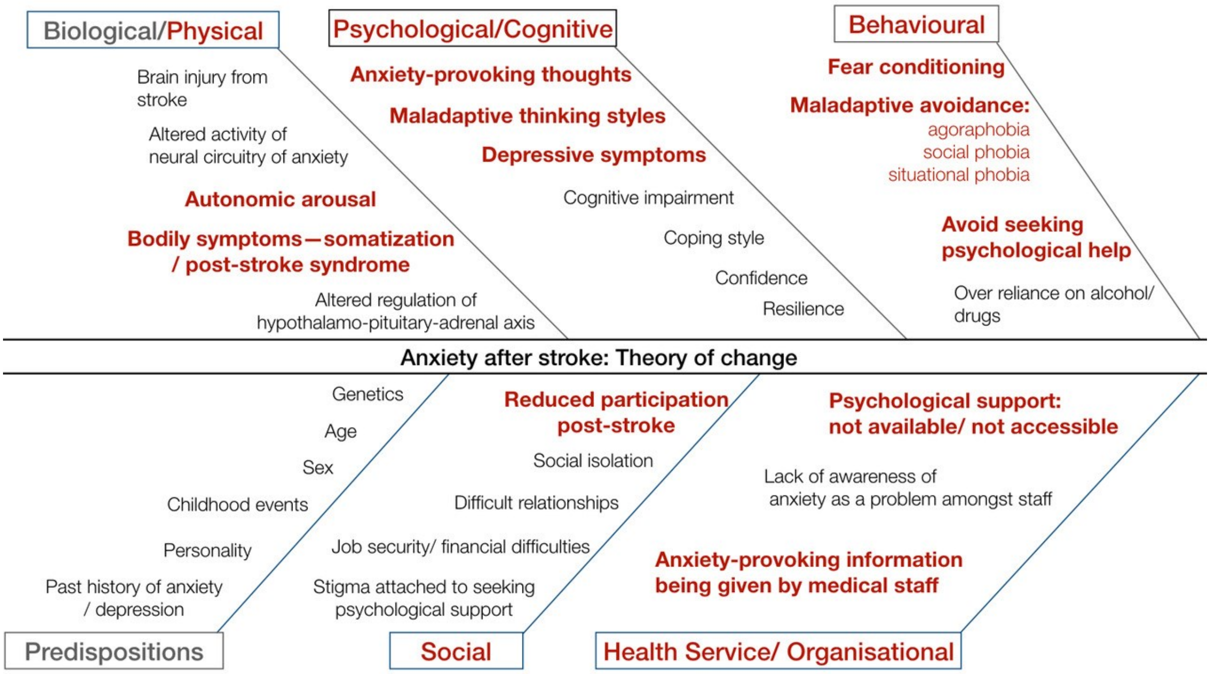

6SQUID Step 4 Theory of action

In conventional CBT, a therapist delivers a course of face-to-face psychotherapy sessions of varying length between 6 weeks to 6 months. CBT is structured, time-limited and goal orientated. Patient and therapist work collaboratively towards a defined goal by working through the key components of CBT: i) identify maladaptive thinking and behaviours, ii) self-monitoring, iii) psychoeducation, iv) cognitive restructuring, v) exposure techniques, vi) specific skills training (varies depending on patient's needs e.g. problem solving, time management), and vii) ongoing self-management[30].

Feasibility of CBT post-stroke

Delivering CBT in a conventional format to all patients with anxiety post-stroke/TIA is unlikely to be feasible in real-world practice given the high prevalence of anxiety in this population, geographical variation in staffing, and other barriers to psychological therapy. A 'theory of action' is required to find a feasible way to deliver the 'active ingredients' of CBT to stroke and TIA patients. The theory of action in Step 4 of the 6SQUID mandates that researchers must think about what resources or assets are available, what is feasible and what is acceptable and ethical in the given context. Adaptations to an intervention (e.g. conventional face-to-face CBT) may be necessary to ensure the intervention's feasibility, acceptability and sustainability at all stages of the future evaluation process and its eventual implementation. Failure to consider this step early could lead to a waste of resources and costly evaluation of an intervention not fit for implementation in the real world. Here we summarize the current context of psychological care post-stroke in the UK, and ways in which key stakeholders' input helped us determine the final design of the TASK intervention.

Barriers in accessing psychological care post-stroke

Inadequate service provision in psychological care post-stroke across the United Kingdom is evident from the reports of the Sentinel Stroke National Audit Programme in England, Wales

and Northern Ireland[31], and the latest Royal College of Physicians Stoke Guideline[32]. The Scottish Stroke Improvement Programme highlighted access to psychological care as a priority area for improvement in 2017[33]. The demand for better access to psychological care post-stroke has consistently been echoed by surveys and qualitative research of patients, carers and health professionals, and through charitable organisations representing stroke patients[34-36].

Stakeholders input I: Scottish Stroke Improvement Programme meeting with the national and local clinical leads in clinical psychology

A workshop was held to discuss the priority set by the Scottish Government's Stroke Improvement Plan to improve delivery of psychological support post-stroke in September 2017. Two of our authors (HYC and MD) attended, one of whom was also the chair of the Scottish Stroke Care Audit (MD). The meeting was chaired and facilitated by the lead of the SIP, and attended by the local leads of clinical psychology services from across Scotland. All agreed that psychological care had to be provided to a large population of stroke patients who needed it—a quarter of stroke patients with anxiety equated to an estimated 2000 to 3000 patients per year in Scotland[33]. All agreed that this demand was unlikely to be met by primary care providers alone, nor the traditional paradigm of referring patients for face-to-face psychotherapy delivered by highly-trained specialists. The shortage and geographical variation in the supply of highly trained psychotherapists in Scotland were evident from the local service evaluations presented at the meeting. These were unlikely to be resolved in the foreseeable future within the National Health Service (NHS). The stepped care model of psychological care, one that has been advocated by the NHS and the Royal College of Physicians Stroke Guideline, was felt to be the most feasible model to provide psychological care post-stroke[37, 38]. An allied health professional with stroke experience e.g. stroke nurse, delivering low-intensity psychological intervention within the lower levels (Level 1 and 2) of this stepped care model, receiving appropriate supervision by a specialist e.g. a consultant clinical psychologist,

was considered a feasible option. Severe or refractory cases would be escalated to the top level of this stepped care model (Level 3) to receive high-intensity psychological intervention from a specialist. Other potential barriers to psychological support specific to stroke patients were also discussed at this meeting: the inability to attend appointments due to physical immobility; reluctance to attend sessions due to agoraphobia (a disproportionate fear of leaving one's house); lack of awareness or recognition of anxiety as a problem necessitating treatment due to cognitive deficits; dysphasia; stigma attached to receiving 'psychological' care.

Stakeholder input II: Survey of 'mode of delivery' in stroke patients with anxiety disorder

We administered a survey in 27 community-based participants who were diagnosed with anxiety disorder at three months post-stroke/TIA in our prospective cohort. The survey (Additional file 5) showed an overwhelming preference (24/27, 89%) for a guided intervention rather than unguided self-help; most preferred having treatment at home or had no preference (21/27, 78%); two thirds did not mind whether the intervention was delivered face-to-face or by telephone (18/27, 66%) ; over two thirds found it acceptable to use online materials as part of the intervention (17/27, 63%); over two thirds were capable of using the internet with or without help (20/27, 70%).

Design of TASK-CBT intervention

Centralized delivery

We designed a centralized model to deliver an individualised CBT-based intervention via telemedicine. Being able to deliver this intervention remotely makes it feasible to centralize the personnel required. This serves to overcome the geographical variation and chronic shortage in workforce that is likely to continue. This model of delivery offers the potential of delivering the intervention as widely, and as efficiently as possible across the country, both in a large-scale RCT, and ultimately in real-world clinical practice.

Individualized guided-CBT

Therapeutic alliance between the therapist and patient is an integral part of conventional CBT and is preserved in our TASK-CBT. This alliance was clearly valued by our surveyed patients, clinical psychologists and psychiatrists. RCT evidence to-date demonstrated that guided self-help CBT interventions were more efficacious than self-help only (bibliotherapy or computerized)[39, 40].

Non-specialist stroke health professional to deliver TASK-CBT—a low intensity CBT-based psychological intervention

In line with the views of the stakeholders, we designed our TASK-CBT intervention to be delivered by an appropriately trained health professional with experience working with stroke patients e.g. stroke nurse, stroke physician, stroke rehabilitation therapists under supervision of a specialist e.g. psychiatrist or clinical psychologist. The TASK-CBT intervention represents a low-level psychological intervention (steps 1 and 2) in the stepped care model[39].

Patient involvement in co-producing the content of TASK-CBT intervention

We conducted a patient involvement group to co-produce the content of TASK-CBT intervention, reported in Additional file 5. Based on their input, we included 'patient stories' on the treatment website to illustrate the common anxiety problems faced by stroke survivors. A disproportionate fear of stroke recurrence and associated maladaptive behaviours are key targets for our TASK-CBT intervention. We included video and educational content on 'Bodily symptoms: another stroke or anxiety?' in the TASK-CBT website.

Modelling processes of the TASK-CBT intervention

Following the 6SQUID approach we summarize the programme theory (theory of change + theory of action) of the TASK-CBT development in a logical model diagram (Figure 3 of main paper).

6SQUID Step 5 Testing and refining the intervention in a small scale

The current TASK feasibility RCT reported in the main paper represents the initial feasibility testing and process evaluation of the TASK intervention. Further refinements of the TASK-CBT intervention are likely needed prior to definitive evaluation in a large randomized controlled trial.

References

1. Wight D, Wimbush E, Jepson R, Doi L. Six steps in quality intervention development (6squid). *Journal of epidemiology and community health*. 2016;70:520-525
2. Stroke association, uk. State of the nation stroke statistics - january 2017.
3. Chun HY, Whiteley WN, Dennis MS, Mead GE, Carson AJ. Anxiety after stroke: The importance of subtyping. *Stroke*. 2018
4. DSM-V. Diagnostic and statistical manual of mental disorders (5th edition) (dsm-v). American psychiatric association. Arlington, va: American psychiatric publishing. 2013. 2013
5. Campbell Burton CA, Murray J, Holmes J, Astin F, Greenwood D, Knapp P. Frequency of anxiety after stroke: A systematic review and meta-analysis of observational studies. *International journal of stroke : official journal of the International Stroke Society*. 2013;8:545-559
6. Ayerbe L, Ayis SA, Crichton S, Wolfe CD, Rudd AG. Natural history, predictors and associated outcomes of anxiety up to 10 years after stroke: The south london stroke register. *Age Ageing*. 2014;43:542-547
7. Wolitzky-Taylor KB, Horowitz JD, Powers MB, Telch MJ. Psychological approaches in the treatment of specific phobias: A meta-analysis. *Clinical psychology review*. 2008;28:1021-1037
8. Cuijpers P, Sijbrandij M, Koole S, Huibers M, Berking M, Andersson G. Psychological treatment of generalized anxiety disorder: A meta-analysis. *Clinical psychology review*. 2014;34:130-140
9. Stein MB, Sareen J. Clinical practice. Generalized anxiety disorder. *The New England journal of medicine*. 2015;373:2059-2068
10. Charney DS, Grillon C, Bremner JD. Review : The neurobiological basis of anxiety and fear: Circuits, mechanisms, and neurochemical interactions (part i. *The Neuroscientist*. 1998;4:35-44
11. Etkin A, Wager TD. Functional neuroimaging of anxiety: A meta-analysis of emotional processing in ptsd, social anxiety disorder, and specific phobia. *The American journal of psychiatry*. 2007;164:1476-1488
12. Hofmann SG, Smits JAJ. Cognitive-behavioral therapy for adult anxiety disorders: A meta-analysis of randomized placebo-controlled trials. *The Journal of clinical psychiatry*. 2008;69:621-632
13. Pavlov IPG, W. Horsley; Volborth, G.; and Cannon, Walter B. Lectures on conditioned reflexes twenty-five years of objective study of the higher nervous activity (behaviour) of animals. *Historical Medical Books. 35.* 1928
14. Watson JBR, R. Conditioned emotional reactions. *Journal of Experimental Psychology*. 1920;3:1-14

15. Jones MC. A laboratory study of fear: The case of peter. *First published in Pedagogical Seminary*. 1924;31:308-315
16. Skinner BF. Science and human behavior. The b.F. Skinner foundation. 1953
17. Lindsley OR. Operant conditioning methods applied to research in chronic schizophrenia. *Psychiatric Research Reports*. 1956;5:118-139
18. Wolpe J. The systematic desensitization treatment of neuroses. *The Journal of nervous and mental disease*. 1961;132:189-203
19. Hofmann SG, Asnaani A, Vonk IJ, Sawyer AT, Fang A. The efficacy of cognitive behavioral therapy: A review of meta-analyses. *Cognit Ther Res*. 2012;36:427-440
20. Chun HY, Newman R, Whiteley WN, Dennis M, Mead GE, Carson AJ. A systematic review of anxiety interventions in stroke and acquired brain injury: Efficacy and trial design. *J Psychosom Res*. 2018;104:65-75
21. Beck AT. Cognitive therapy: Nature and relation to behavior therapy. *Behavior Therapy*. 1970;1:184-200
22. Chun HYW, W.; Mead, G.; Dennis M.; Carson, A. Anxiety after stroke: The importance of subtyping. *Stroke*. 2018;(in press)
23. Salter K, Hellings C, Foley N, Teasell R. The experience of living with stroke: A qualitative meta-synthesis. *Journal of rehabilitation medicine*. 2008;40:595-602
24. Woodman P, Riazi A, Pereira C, Jones F. Social participation post stroke: A meta-ethnographic review of the experiences and views of community-dwelling stroke survivors. *Disabil Rehabil*. 2014;36:2031-2043
25. Burton CR. Living with stroke: A phenomenological study. *J Adv Nurs*. 2000;32:301-309
26. Dowswell G, Lawler J, Dowswell T, Young J, Forster A, Hearn J. Investigating recovery from stroke: A qualitative study. *J Clin Nurs*. 2000;9:507-515
27. Menlove L, Crayton E, Kneebone I, Allen-Crooks R, Otto E, Harder H. Predictors of anxiety after stroke: A systematic review of observational studies. *Journal of Stroke and Cerebrovascular Diseases*. 2015;24:1107-1117
28. Wright F, Wu S, Chun HY, Mead G. Factors associated with poststroke anxiety: A systematic review and meta-analysis. *Stroke research and treatment*. 2017;2017:2124743
29. Tan-Kristanto S, Kiropoulos LA. Resilience, self-efficacy, coping styles and depressive and anxiety symptoms in those newly diagnosed with multiple sclerosis. *Psychology, health & medicine*. 2015;20:635-645
30. Hambrick JPC, J. S.; Albano, A. M. Chapter 18 cognitive-behavioral treatment of anxiety disorders: Model and current issues. 'Anxiety disorders: Theory, research, and clinical perspectives'. Cambridge university press. New york. 2010:204-209
31. Royal college of physicians intercollegiate stroke working party. Sentinel stroke national audit programme (ssnap). Acute organisational audit report. November 2016. National report on england, wales and northern ireland. 2016
32. RCP. Royal college of physicians national guideline for stroke fifth edition, 2016. The intercollegiate stroke working party. 2016
33. NHS. Scottish stroke improvement programme report, scottish stroke care audit. Nhs scotland. 2017
34. Harrison M, Ryan T, Gardiner C, Jones A. Psychological and emotional needs, assessment, and support post-stroke: A multi-perspective qualitative study. *Topics in stroke rehabilitation*. 2017;24:119-125
35. Morris R. Meeting the psychological needs of community-living stroke patients and carers: A study of third sector provision. *Disability and Rehabilitation*. 2016;38:52-61
36. Stroke association 'feeling overwhelmed': The emotional impact of stroke. Life after stroke campaign report. . 2013
37. NICE. Common mental health problems: Identification and pathways to care. Nice clinical guideline [cg123]. 2011

38. Gillham SC, L. Nhs improvment-stroke -psychological care after stroke--improving stroke services for people with cognitive and mood disorders. 2011
39. Lewis C, Pearce J, Bisson JI. Efficacy, cost-effectiveness and acceptability of self-help interventions for anxiety disorders: Systematic review. *The British journal of psychiatry : the journal of mental science*. 2012;200:15-21
40. Olthuis JV, Watt MC, Bailey K, Hayden JA, Stewart SH. Therapist-supported internet cognitive behavioural therapy for anxiety disorders in adults. *The Cochrane database of systematic reviews*. 2015:Cd011565
