# Supplementary material for: Treating anxiety after stroke (TASK): the feasibility phase of a novel web-enabled randomised controlled trial
Source: Pilot Feasibility Stud. 2018 Aug 14;4:139. doi: 10.1186/s40814-018-0329-x (PMC6092858; doi:10.1186/s40814-018-0329-x)
Supplement: Supplementary file 2 — Report of patient involvement in TASK. (DOCX 463 kb) [file 40814_2018_329_MOESM2_ESM.docx]

**Additional file 2**

**Report of patient involvement in the design of TASK (Treating Anxiety after StroKe)**

**Aim**:

To involve stroke patients who have experienced anxiety after stroke in order to optimize the relevance and acceptability of our intervention and randomized controlled trial design.

**Methods:**

1. A quantitative survey of patients on anxiety intervention design and mode of delivery
2. A patient advisory group meeting to co-design the content and treatment materials

i) **A quantitative survey on intervention design and delivery**

This was carried out as part of our recent prospective cohort study^1^. At the end of the semi-structured interviews I invited participants who reported anxiety problems to take part in this additional survey. 27 out of 49 participants were able to complete this survey. The remainder declined due to the feelings of tiredness, fatigue and inability to continue with more questioning.

I administered nine-questions with set responses to choose from (Table 1), on aspects of intervention design. These were patient preferences on i) guidance, ii) mode of guidance iii) mode of delivery of treatment content iv) frequency of contact, v) treatment venue, vi) time to start treatment course. I recorded any additional comments related to intervention design and delivery.

Table 1**.** Questions on intervention delivery and set responses for selection

| **If we were going to give you a treatment program to help with your anxiety……** | |
| --- | --- |
|  | **Choice of responses** |
| **1) Would you prefer having it with or without guidance?** | ‘prefer with guidance’  ‘prefer self-help with no guidance’ |
| **2) How would you like to receive the guidance?** | ‘face-to-face’  ‘telephone’  ‘don’t mind—either or both is fine’  ‘none’ |
| **3) Would you find it acceptable to use online resources/ materials as part of your treatment?** | ‘yes’  ‘no’ |
| **4) Would you find it acceptable to use a handbook as part of your treatment?** | ‘yes’  ‘no’ |
| **5) Are you able to use the internet?** | ‘yes’  ‘yes with help’  ‘no’ |
| **6) Would you accept having family involved in your treatment?** | ‘yes’  ‘no’  ‘maybe’ |
| **7) Where would you prefer to have your treatment?** | ‘at home’  ‘at clinic’  ‘no preference’ |
| **8) How often would you like to be contacted? (see comments)** | ‘once per week’  ‘once every two weeks’  ‘once every three weeks’  ‘no contact at all’ |
| **9) When should the treatment program start?** | ‘within first month’  ‘within first two months’  ‘at three months or later’ |
|  |  |

**ii) A patient advisory group (PAG) meeting to co-design the content and treatment materials**

There were two main objectives for this first PAG meeting:

1. To hear the perspectives of patients who actually experienced anxiety after stroke on treatment content and design of treatment materials
2. To hear the patients’ insights into how losses to follow-up in a randomized controlled trial could be minimized

*Selection of patients for the PAG*

In my prospective cohort study I recorded a list of participants who consented to be contacted again for future involvement in the design of my anxiety intervention. I selected those who were diagnosed with an anxiety disorder on SCID.

In total, there were 20 participants whom I could contact. I aimed to invite around eight people to take part in this PAG.

A letter of invitation along with an event programme (Figure 1) was sent to 10 people, followed by a telephone call the following week. I sent the remaining 10 invitations two weeks later based on the number of responses received from the first batch of invitations. I confirmed their intention to attend by telephone one week ahead of the PAG meeting.

*Structure and topic for the PAG*

In designing the structure and topic guides for this PAG, I referred to the ‘INVOLVE Briefing notes for researchers: public involvement in NHS, public health and social care research’^2^. I used a set of PowerPoint slides to guide the patient advisors through the PAG meeting. There were three open discussions. The topics and prompts are listed below in Table 2.

Figure 1. Programme for the PAG meeting, sent alongside the invitation letter


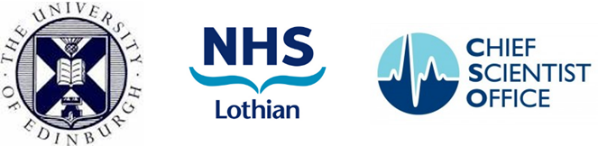


**Date:** 6^th^ April, 2017

**Time:** 2:00 – 4:15 pm

**Venue:** Chancellor’s Building, Royal Infirmary of Edinburgh

**No. of participants:** 8 (expected to attend)

**Programme**

2:00 - 2:10 **Welcome and introduction**

2:10 – 2:20 **Presentation**: What did we learn from our research project last year?

2:20 – 3:00 **Open discussion**:

What made you anxious after your stroke or TIA?

What help, if any, were you able to get?

What would you have wanted in your treatment for anxiety?

3:00 – 3:10 **Break**

Have a browse at some existing booklets and websites

3:10 – 3:30 **Open discussion:** treatment content and design

What would you find helpful?

3:30 – 3:40 **Presentation:** Why is testing treatment important?

3:40 – 4:00 **Open discussion:**

How can we get participants to complete the treatment?

What make participants drop out?

How can we get participants to complete follow up?

4:00 – 4.15 **Summary, closing remarks**

Ongoing opportunity to be a ‘patient advisor’

**Table 2.** Topics and prompts for the open discussions at the PAG meeting

| **First open discussion (40minutes)**  **Topic: Support for anxiety problems after stroke**  **Prompts:**  What were your anxiety, worries or fear after your stroke?  What help or support, if any, did you receive?  What would you have wanted from the stroke service? |
| --- |
| **Second open discussion (30minutes)**  **Topic:** **Treatment content and design of treatment materials**  This discussion follows a review of existing resources e.g. leaflets, booklets, the stroke workbook  **Prompts:**  What are your initial thoughts about these materials?  What recommendations do you have for designing the materials for anxiety after stroke?  What messages do you think are most effective? Least effective? |
| **Third open discussion (10minutes)**  **Topic: Why is testing treatment important?**  **Prompts:**  What makes people drop out of a clinical trial?  What can we do to encourage people to:  complete trial treatment?  complete follow-up questionnaire several months after treatment has finished? |

*Personnel*

I chaired the PAG meeting and acted as the facilitator for the open discussions. GEM, a professor of stroke medicine attended and contributed in the open discussions. Two other researchers assisted at the event. One scribed and audio-recorded the open discussions on a digital recorder.

The PAG reviewed the following stroke resources:

1. ‘Feeling overwhelmed—the emotional impact of stroke’, a booklet published by the Stroke Association’s ‘Life After Stroke Campaign’ in the Summer, 2013
2. ‘Living with stress and anxiety’ leaflet F23, September 2013, by Chest Heart & Stroke Scotland
3. A selection of leaflets and booklets by Chest Heart stroke Scotland (CHSS) on other issues e.g. air travel, sex after stroke illness, thinking and behaviour issues after stroke, stroke in younger people
4. ‘Stroke workbook’, a supported self-management programme published by Lothian Health Board, 2011
5. Self-help for stroke website <http://selfhelp4stroke.org/>, developed by CHSS, in partnership with NHS Scotland, the University of Edinburgh and stroke survivors living in Scotland.

*Consent to audio-recording, photography and publication of narrative*

All patient advisors gave verbal consent to have their discussions audio-recorded and photographs taken. All consented to the publication of their narratives anonymously.

*Ethics*

The South East Scotland research ethics committee confirmed no ethical approval was required for this PAG meeting, which was considered a public and patient involvement event.

*Summarizing and reporting of data*

I reviewed all hand-scribed and audio-data and reported the key points raised by our PAG for each topic discussed using their narratives I extracted from the recorded data.

**Results**

*1)* Quantitative survey of 27 patients with anxiety disorder (Figure 2).

| 1) Would you prefer to have it with or without guidance | |
| --- | --- |
| With guidance (24)  Without (3) |  |
| 2) How would you like to receive the guidance? | |
| Either face-to-face or telephone (18)  Face-to-face (6)  Telephone (2)  No contact (1) |  |
| 3) Would you find it acceptable to use online resources/ materials as part of your treatment? | |
| Yes (17)  No (10) |  |
| 4) Would you find it acceptable to use handbook as part of your treatment? | |
| Yes (27)  No (0) |  |
| 5) Are you able to use the internet? | |
| Yes (18)  No (7)  Yes with help (2) |  |
| 6) Would you accept having family involved in your treatment? | |
| Yes (12)  No (12)  Maybe (3) |  |
| 7) Where would you prefer having your treatment? | |
| Home (13)  No preference (8)  Clinic (6) |  |
| 8) How often would you like to be contacted? | |
| Once every 3 weeks (12)  Once per week (8)  Once every 2 weeks (6)  No contact at all (1) |  |
| 9) When should treatment program begin? | |
| Within the first month of stroke (25)  Within the first 2 months (2)  At 3 months or later (0) |  |

**Additional comments made by survey participants**

**A 56 year-old,**

***“****I needed more information on discharge on the emotional consequences after stroke. I felt safe in the hospital but once I got home I felt alone. Other people didn’t understand why I felt so severely affected (emotionally) by my stroke. I didn’t look disabled”*

**An 84 year-old,**

*“It would have been good to have spoken to someone about my anxiety to find that it was not uncommon”*

**A 65 year-old,**

*“I think I need guidance as I won’t know what I’m doing. I don’t mind what kind of contact. I had anxiety pretty much straight after my stroke.”*

**A 62 year-old,**

“*I felt someone should have at least raised the issue about anxiety within the first month of my diagnosis”*

**A 60 year-old,**

*“I would have liked the support at one month after stroke. That was when I tried to return to normal living.”*

**ii) Narratives from Patient Advisory Group Meeting on 6^th^ April, 2017**

| **Discussion 1) Support for anxiety problems after stroke** | |
| --- | --- |
| **Prompts:**  What were your anxiety, worries or fear after your stroke?  What help or support, if any, did you receive?  What would you have wanted from the stroke service?  Further comments on what they would find helpful | **Patient Advisor 1 (age 73)**  *“It was a total shock for me. I just didn’t understand why I got a stroke when I was the fit one. My first anxiety was that it was going to happen again”*  *“I was afraid to go out for some time. I had this horrible vision that something awful would happen to me if I took the train”*  *“It took me a long time even to get on the bus to visit my daughter. I was afraid of something happening.”*  *“My daughter was getting married not long after my stroke so I knew I had to turn up. So I did it and that was that!”*  *“I thought to myself I could either sit here or get a grip. I told myself not to be silly”*  **Patient Advisor 2 (age 72)**  ***“****The anxiety was about leaving the house.* *The first six weeks were very difficult. When I took the bus or drove my car I just wanted to get back home.”*  *“A flash in the head would make me wonder—oops is there something wrong?”*  *“It took a lot of effort. It took about 6 weeks before I could do a trip to London and to stay overnight. I had to work quite hard to persuade myself that I had to do it. I succeeded and so just got on with it.”*  *“I think we were all* (**Patient advisors 1 and 3 agreed**) *dreading the thought of being caught ill in a public place, and the total loss of control. That is the greatest fear of all.”*  *“Living alone meant that one just had to get on with it! No choice in the matter, still got work to do. No point sitting here twiddling my fingers. Forget yesterday and think about tomorrow.”*  **Patient Advisor 3 (age 62)**  *“I was still very wobbly on my feet. I had other medical problems already and the stroke was another thing to add to my list.”*  **Patient Advisor 1:**  *“I did not receive any help. Nobody asked me how I was.”*  **Patient Advisor 2:**  *“I was visited by the Chest Heart Stroke Scotland community nurse at home. I didn’t think it would be of any use initially, but he turned out to be excellent. I found him extremely helpful. He gave me a lot of information. We chatted for an hour. He offered contact details in case I needed anything”*  **Patient Advisor 3:**  *“I found the biggest support was by going to the community stroke service”*  *“I found being amongst other people in the same situation really helped, to know that I was not odd.”*  *“It was easier to talk to strangers who had similar experience than talking about it at home.”*  *“They gave me confidence to try things”*  **Patient Advisor 1:**  *“I was just told everything was going to be fine but of course that was not the case.”*  *“I would have liked more information given to me after discharge”*  **Patient advisor 2:**  *“it’s surprising that there is no follow up after a stroke…while there was follow up for my heart attack and after my hip operation”*  **Patient advisor 3:**  *“I would have liked some information on what services would be available after discharge?”*  *“I only stumbled upon the stroke community centre by accident”*  **Patient advisor 1**  *“being told not to worry is not terribly helpful”*  **Patient advisor 2**  *“you have got to have something in place, have a list of emergency numbers in your wallet, and on your desk at home (if you live alone)”*  *“I never leave my house without my mobile phone in my pocket”*  *“for people who live alone, I would recommend a pendant alarm (pointing at his) and a note with emergency contact details”*  *“A patient group will not work for me but it does for other people so I guess it depends on personal preference”* |
| **Discussion 2: Treatment content and design of treatment materials**  This discussion follows a review of existing resources e.g. leaflets, booklets, the stroke workbook | |
| **Prompts:**  What are your initial thoughts about these materials?  What recommendations do you have for designing the materials for anxiety after stroke?  What messages do you think are most effective? Least effective? | Referring to the Stroke Association’s ‘Feeling overwhelmed’ booklet  **Patient advisor 2**  *“this is excellent and it is most interesting reading about other people’s experiences”*  **Patient advisor 3**  *“It is very valuable to learn from other people’s experiences and how stroke affects other people. It makes me less afraid to talk about things that I would otherwise tend to shy away from…”*  **Referring to information leaflets and website**  **Patient advisor 1**  *“I would like information explaining this (stroke) is what happened to you, we don’t know what caused it but these are the things that could help”*  *“Acknowledging uncertainty is important”*  *“I like the use of pictures—it’s a way of learning things. Also the use of boxes—can really grab attention”*  *“there is so much on the internet, it’s hard to know what is reliable”*  **Patient advisor 2**  *“I would highlight these particular sentences in a different colour—****learn how to recognize anxiety symptoms, you may often mistake your symptoms for illness….****this was absolutely key to what my symptoms were (I had odd sensations, headache)”*  *“The busier you are the better. When I was busy I never noticed any of these symptoms”*  *“it was important to keep myself both mentally and physically active”*  **Patient advisor 3**  *“any information is valuable”*    *“I also found some exercises from a website to help with my balance”*  **Referring to the Stroke Workbook**  **Patient advisor 1**  *“Wouldn’t work at all for me. I already have the answers to what’s in the book. It is not going to alleviate any stress I have. I need something to banish what’s on my mind—the thoughts that things are going to happen to me again”*  **Patient advisor 3**  *“I personally quite like reading this. I like the pictures. It is an easy way for me to learn things”*  *“I think a diary is very boring”*  **Additional comments:**  *“Language needs to be simple and unpatronising”*  *“A book with too much text makes people think they have to sit down properly to read it. With leaflets, you can just pick it up to have a read.”*  *“need positivity all the way through, try to get people with anxiety to think about what is stopping them from returning to their activities, then get them to think about how they used to enjoy these activities, and the positive things that came out of it”*  *“for people who avoid social situations—it may help to tell other people that you have had a stroke” “if they don’t know, they can’t help you or understand you”* |
| **Topic: Why is testing treatment important?** | |
| **Prompts:**  What makes people drop out of a clinical trial?  What can we do to encourage people to:  complete trial treatment?  complete follow-up questionnaire several months after treatment has finished? | **Patient advisor 3:**  *“people are always more enthusiastic at the beginning”*  **Patient advisor 1:**  *“there could be so many reasons, you’d probably have to ask the people who did not turn up to this meeting today and ask them why in a non-confrontational manner”*  **Patient advisor 1:**  *“establish people’s expectations—often our expectations are very high for the trial treatment”*  *“make us feel like we are all involved in the trial, and not just merely finishing a trial treatment”*  *“make people realise there is an end goal—we want to know the results of any research we take part in”*  *“tell us how our participation helps contribute to developing better treatment/care for other people, and how you will use this research”*  *“emphasize on our participation and our involvement in the research instead of using the words like ‘treatment’ or ‘intervention’--feels like something is being done to you”*  **Patient advisor 2:**  *“incentivise people by explaining that there will be something valuable yielded at the end. I was personally very fascinated to see the paper at the end of this cardiac MRI study I took part in”* |

**Summary of report**

***Mode of delivery, timing and frequency***

An anxiety intervention would be most acceptable if patients with anxiety could receive it at home, with guidance via the telephone and the use of either or both, online and written materials. The intervention should preferably commence within the first two months of diagnosis. Anxiety problems appeared to start shortly after being discharged home, as patients tried to return to their normal living.

***Adapting the TASK content based on patient involvement***

- 1. *Our patient advisors expressed that it would be helpful to read about other patients’ experiences with anxiety after stroke*
     - **What we did:** We anonymized and edited 4 narrative accounts from the interviews in our prospective cohort to be displayed as ‘patient stories’ online, alongside a link to the Stroke Association UK’s public document on ‘Feeling overwhelmed’*.*
  2. *Our patient advisors said stroke recurrence was the most feared consequence and had to be addressed*
     - **What we did:** our observational data was consistent with this suggestion. CBT techniques to counteract the disproportionate fear of stroke recurrence and associated maladaptive behaviour are key topics in our TASK-CBT intervention
  3. *Our patient advisors felt it was important to explore the individual’s rationale behind their avoidant behaviour, weighing up the positive gains of changing such behaviour against the losses caused by continual avoidance*
     - **What we did:** the patient advisors were in fact describing a core part of CBT—cognitive restructuring, which is also the most emphasized part of our TASK-CBT intervention
  4. *Our patient advisors would like information to highlight the symptoms of anxiety, especially the bodily sensations that might be mistaken for illness e.g. pain, headache, ‘twinge’. This was the main trigger that perpetuated one of the patient advisor’s anxiety following his stroke.*
- **What we did:** Our TASK-CBT includes a video on ‘Bodily symptoms: another stroke or anxiety?’, and will be one of the individualised target of TASK-CBT


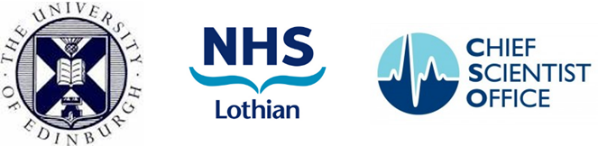


**Invitation to take part in a focus group event on ‘Treating anxiety after stroke and TIA’—6^th^ April 2017**

**3/3/2017**

Dear [title] [surname],

Thank you for taking part in my research project on ‘Anxiety after stroke’ last year.

Your participation in research has been immensely helpful to my project.

I hope to invite you to help us design a treatment program to help people’s anxiety following a stroke.

I am holding a focus group event to gather opinion from people with experiences of a stroke or mini-stroke.

The event will take an afternoon.

There will be open discussions on how to improve the design of our treatment.

Please find enclosed a provisional programme for the vent.

I will be in touch with you by telephone in the coming weeks to see if you would like to take part in this discussion group.

If you feel you cannot take part in the focus group but would like to help me design anxiety treatment let me know and I can make alternative arrangements.

Feel free to contact me on:

Mobile:

Email: [ho-yan.chun@nhslothian.scot.nhs.uk](mailto:ho-yan.chun@nhslothian.scot.nhs.uk)

Or by post via the pre-paid envelope.

Yours sincerely,

Dr Yvonne Chun

Clinical researcher and stroke doctor

Dr Chun will be contacting you by telephone anyway.

You can also reply by using this return slip and the pre-paid envelope

**Return slip for focus group on Thursday 6^th^ April, 2017**

I will take part in the focus group meeting on 6^th^ April, 2017

I will not be attending the focus group event on 6^th^ April, 2017

I would like to be involved in the design of the treatment programme anyway

Your name: ____________________________

**References**

1. INVOLVE (2012) Briefing notes for researchers involving the public in NHS, public health and social care resaerch. INVOLVE, Eastleigh

1. Chun HY, Whiteley WN, Dennis MS, Mead GE, Carson AJ. Anxiety after stroke: The importance of subtyping. *Stroke*. 2018

2. Involve briefing notes for researchers <http://www.Invo.Org.Uk/posttypepublication/involve-briefing-notes-for-researchers/>.
