# Supplementary material for: Treating anxiety after stroke (TASK): the feasibility phase of a novel web-enabled randomised controlled trial
Source: Pilot Feasibility Stud. 2018 Aug 14;4:139. doi: 10.1186/s40814-018-0329-x (PMC6092858; doi:10.1186/s40814-018-0329-x)
Supplement: Supplementary file 5 — Screening questions in ‘Sign up and consent form’. (PDF 173 kb) [file 40814_2018_329_MOESM5_ESM.pdf]

Resize font: + | −

Disable speech

TASK Treating Anxiety after StroKe

Research team contact details
Chief investigator: Dr Yvonne Chun (stroke doctor and clinical research fellow)
TASK research team mobile: 0745 320 7061
TASK research team email: task.trial@ed.ac.uk

Treating Anxiety after Stroke (TASK) study sign-up form

Trouble reading? Click on the speaker button on the left hand side of each question

(TASK_ICF_v3 1.12.2017)

(A) Eligibility checklist

Please answer these questions to check you are eligible to take part in the TASK study

1) During the past 2 weeks I have been bothered by worrying too much about different things

* must provide value

Yes | No | reset

Have you avoided the following situation(s) because of fear or other unpleasant feelings?

2) walking alone in busy streets

* must provide value

Yes | No | reset

3) going into crowded shops

* must provide value

Yes | No | reset

4) eating or drinking with other people

* must provide value

Yes | No | reset

5) any of your normal day-to-day activities for fear of having a headache (or other odd sensations)

* must provide value

Yes | No | reset

6) If you answered 'no' to all of the above Q1-5, what anxiety problem(s) are you experiencing?
