# Supplementary material for: Treating anxiety after stroke (TASK): the feasibility phase of a novel web-enabled randomised controlled trial
Source: Pilot Feasibility Stud. 2018 Aug 14;4:139. doi: 10.1186/s40814-018-0329-x (PMC6092858; doi:10.1186/s40814-018-0329-x)
Supplement: Supplementary file 6 — Research ethics approval letter. (PDF 134 kb) [file 40814_2018_329_MOESM6_ESM.pdf]

**South East Scotland REC 02**
2 - 4 Waterloo Place
Edinburgh
EH1 3EG

Telephone: 0131 465 5674
Fax:

18 December 2017

Dr Ho-Yan Yvonne Chun
Clinical research fellow
University of Edinburgh
Centre for Clinical Brain Sciences
Chancellor's Building, 49 Little France Crescent
Edinburgh
EH16 4SB

Dear Dr Chun

| | |
|---|---|
| **Study title:** | **Treating Anxiety after StroKe (TASK)â€"feasibility randomized controlled trial** |
| **REC reference:** | **17/SS/0143** |
| **Protocol number:** | **AC17087** |
| **IRAS project ID:** | **228995** |

Thank you for your letter of 5th December 2017. I can confirm the REC has received the documents listed below and that these comply with the approval conditions detailed in our letter dated 24 November 2017

**Documents received**

The documents received were as follows:

| *Document* | *Version* | *Date* |
|---|---|---|
| Covering letter on headed paper | | 05 December 2017 |
| GP/consultant information sheets or letters [GP letter] | 3 | 01 December 2017 |
| Non-validated questionnaire [participant feedback screenshots] | | |
| Other [electronic PIS screenshots] | | |
| Other [the TASK electronic ICF v3_1.12.17] | | |
| Participant consent form [participant consent form (paper)] | 3 | 01 December 2017 |
| Participant information sheet (PIS) [paper PIS] | 3 | 01 December 2017 |
| Research protocol or project proposal [TASK Protocol] | 3 | 01 December 2017 |
| Response to Additional Conditions Met [Response to additional conditions met cover letter] | | 05 December 2017 |

**Approved documents**

The final list of approved documentation for the study is therefore as follows:

| *Document* | *Version* | *Date* |
|---|---|---|
| Copies of advertisement materials for research participants [Links to TASK websites ] | 1 | 10 October 2017 |
| Costing template (commercial projects) [Funding source] | | |
| Covering letter on headed paper | | 05 December 2017 |
| Evidence of Sponsor insurance or indemnity (non NHS Sponsors only) [Professional indemnity] | | 04 August 2017 |
| GP/consultant information sheets or letters [GP letter] | 3 | 01 December 2017 |
| IRAS Application Form [IRAS_Form_16102017] | | 16 October 2017 |
| Letters of invitation to participant [Letter from treating physician] | 2 | 10 October 2017 |
| Non-validated questionnaire [participant feedback screenshots] | | |
| Other [public liability insurance University of Edinburgh] | | |
| Other [electronic PIS screenshots] | | |
| Other [the TASK electronic ICF v3_1.12.17] | | |
| Participant consent form [participant consent form (paper)] | 3 | 01 December 2017 |
| Participant information sheet (PIS) [paper PIS] | 3 | 01 December 2017 |
| Research protocol or project proposal [TASK Protocol] | 3 | 01 December 2017 |
| Response to Additional Conditions Met [Response to additional conditions met cover letter] | | 05 December 2017 |
| Summary CV for Chief Investigator (CI) [CV of CI] | 1 | 14 September 2017 |
| Summary of any applicable exclusions to sponsor insurance (non-NHS sponsors only) [clinical trial liability insurance] | | 27 July 2017 |
| Validated questionnaire [questionnaire set including mRS, EQ5D5L-VAS, PHQ2, GAD7, and FQ] | | 18 October 2017 |
| Validated questionnaire [Follow up surveys screenshots] | | |

You should ensure that the sponsor has a copy of the final documentation for the study. It is the sponsor's responsibility to ensure that the documentation is made available to R&D offices at all participating sites.

| **17/SS/0143** | **Please quote this number on all correspondence** |
|---|---|

Yours sincerely

**Joyce Clearie**
**SESREC 2 Manager**

E-mail: joyce.clearie@nhslothian.scot.nhs.uk

*Copy to: , NHS Lothian Research and Development Office*
